# Evaluating the Accuracy of a Vision‐Based Algorithm for Groundline Estimation in Trotting Horses Using Multiple Camera Angles

**DOI:** 10.1002/vms3.70739

**Published:** 2025-12-30

**Authors:** Karsten Key, Katja Berg, Jakob Kirkegaard, Kristian Ringkjær Andresen, Sabrina Skov Hansen

**Affiliations:** ^1^ KeyDiagnostics Fredensborg Denmark; ^2^ iKeyVet Fredensborg Denmark; ^3^ Department of Veterinary Clinical Sciences University of Copenhagen Copenhagen Denmark

**Keywords:** deep learning, equine lameness, groundline estimation, handheld, lameness detection, objective gait analysis, pose estimation, vision‐based algorithm

## Abstract

**Background:**

Equine lameness diagnosis largely relies on subjective visual assessments, which can be biased. Although marker‐based methods, force plates and inertial measurement units (IMUs) provide objective measurements, they require specialized setups. Vision‐based algorithms offer a portable, markerless alternative, but their accuracy needs thorough testing.

**Objectives:**

To evaluate a custom vision‐based algorithm for estimating the groundline across multiple camera angles, including handheld use in horses trotting on a treadmill.

**Study design:**

Experimental comparative study.

**Methods:**

Eight Standardbred trotter mares were recorded trotting on a high‐speed treadmill using seven iPhones positioned at various heights and angles, including a handheld device. A trained deep neural network algorithm placed 2D keypoints on each video frame. Vertical Displacement Signals (VDS) for the eye, withers and croup (tuber sacrale) were computed relative to either an algorithm‐estimated or a fixed treadmill groundline. Maximum (Maxdiff) and minimum (Mindiff) stride values were compared using Bland–Altman analysis, scatter plots and histograms. The effect of handheld use on variability and accuracy was assessed by comparing results from a handheld camera to those from a static camera.

**Results:**

Groundline estimation closely matched the fixed reference, exhibiting near‐zero mean angle error and low mean average error (MAE = 0.45°; *n* = 242.192). Maxdiff and Mindiff stride‐level (*n* = 36.981) MAE were 0.5 mm, with clinically acceptable additional variability introduced by handheld use at the trial level (Maxdiff and Mindiff MAE < 1.8 mm; *n* = 357).

**Main limitations:**

Treadmill‐based data and a single breed/coat colour may limit generalizability to other settings.

**Conclusions:**

The vision‐based algorithm accurately estimates the groundline and stride VDS parameters from various camera setups, including handheld. Further validation in diverse environments and against other objective gait analysis systems is recommended.

## Graphical abstract

A markerless, computer vision‐based algorithm (RealHorse) was evaluated for estimating the groundline in trotting horses from multiple camera angles, including handheld recordings, on a treadmill. The algorithm showed high agreement with a fixed reference, with a groundline angle absolute error of 0.45° and stride‐level Maxdiff and Mindiff absolute errors of just 0.5 mm. Handheld use introduced minimal variability, with trial‐level absolute errors under 1.8 mm, supporting its application in field‐based equine gait analysis.

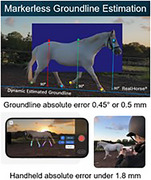

## 1 Introduction

Equine lameness is a significant concern among horse owners and veterinary practitioners, often necessitating objective gait analysis for accurate diagnosis. Traditionally, lameness examination has relied on subjective visual assessment by trained professionals, which can be time‐consuming and expensive (Hardeman, Van Weeren et al. 2022; Keegan 2007). This method, while valuable, is limited by observer bias and variability in the detection of subtle or low‐grade lameness (Arkell et al. 2006; Fuller et al. 2006; Hammarberg et al. 2016; Parkes et al. 2009, Keegan et al. 2010; Hewetson et al. 2006).

To enhance objectivity, various technologies have been developed for gait analysis, including force plates, inertial measurement units (IMUs) and optical motion capture systems (Keegan 2007; Serra Bragança, van Weeren et al. 2018). Force plates provide accurate ground reaction force data but are limited to laboratory settings and can disrupt natural gait (Weishaupt et al. 2004). IMUs, attached to the horse's body, offer portability and use acceleration and angular velocity measurements for gait analysis (Keegan et al. 2011; Pfau et al. 2005). However, the data quality may be affected by sensor placement and attachment stability (Serra Bragança, Wiestner et al. 2018; Keegan et al. 2004; Moorman et al. 2017).

Optical motion capture systems, such as marker‐based systems using multiple high‐speed cameras, provide detailed kinematic data and have been considered the gold standard for kinematic analysis (Hardeman, Egenvall et al. 2022). These systems require reflective markers placed on anatomical landmarks, which can be time‐consuming to set up and may be sensitive to attachment instability and skin motion. Additionally, they are confined to controlled environments due to their reliance on fixed camera setups (Lawin et al. 2023; Wang et al. 2021).

Recent advancements in computer vision and machine learning have enabled the development of algorithms capable of analysing video data from standard smartphones (Lawin et al. 2023; Wang et al. 2021; Pereira et al. 2019), paving the way for marker‐less motion capture using video data. This reduces the need for specialized equipment and allows for more natural movement analysis. The algorithms measure movement symmetry from key anatomical points or body parts, offering a more accessible solution for asymmetry and lameness detection (Lawin et al. 2023). In human biomechanics, deep learning‐based pose estimation models like OpenPose (Cao et al. 2018), DeepPoseKit (Graving et al. 2019) and DeepLabCut (Mathis et al. 2018) have demonstrated high accuracy in tracking body landmarks by comparison with manually annotated keypoints. These models have been adapted for animal studies, including rodent and primate movement analysis (Pereira et al. 2019).

Markerless motion capture is an emerging approach in equine gait analysis, with only a few studies to date demonstrating the potential of deep learning and computer vision in equine biomechanics. (Lawin et al. 2023; Wang et al. 2021; Kallerud et al. 2024).

It is generally recognized that monitoring the cyclical vertical movements of key anatomical landmarks during trot, particularly the head, withers and pelvis, is valuable for evaluating overall gait symmetry and detecting lameness. Of particular significance are the indicators of impact and push‐off asymmetry within each stride cycle, quantified by differences in minimum vertical displacement (Mindiff) and maximum vertical displacement (Maxdiff). Among the many systems for objective gait analysis, some (like the one in this study) derive a vertical displacement signal (VDS) referenced directly or indirectly to the ground (ground truth) (Pfau et al. 2005; Hardeman, Egenvall et al. 2022; Roepstorff et al. 2021; Buchner et al. 1996; Rhodin et al. 2022; Starke and Clayton 2015), while others analyse the relationship between the first and second harmonics of the VDS (Keegan et al. 2011; Keegan et al. 2004; Lawin et al. 2023; Bosch et al. 2018). Referencing the VDS to the ground reduces sensitivity to uneven surfaces, which are commonly encountered in clinical field settings, enabling image stabilization and a conversion from pixel distance to a metric value of the gait symmetry metrics.

To date, limited research has focused on the impact of camera positioning and movement (e.g., handheld vs. stationary) on the reliability of such markerless gait analysis algorithms. Understanding how these factors affect keypoint detection and symmetry measurements is essential when using normal 2D cameras, from varying angles and with handheld use, as seen in field use. Others have explored the use of static camera positions from the front and rear of the horses (Lawin et al. 2023; Wang et al. 2021), however, studies are needed in relation to seeing horses from the side.

This study aimed to evaluate a markerless vision‐based algorithm for estimating the groundline in trotting horses observed from the side, a critical reference for calculating VDS. We investigated the algorithm's accuracy and robustness by comparing estimated groundlines to a fixed groundline across a variety of camera angles and perspectives, including handheld use. Our objective was to assess whether the algorithm could consistently estimate the groundline and generate reliable stride‐level symmetry metrics (Maxdiff and Mindiff) under varied recording conditions. We also evaluated whether handheld video introduces clinically relevant error compared to a stationary camera. Our hypothesis was that the algorithm would produce groundline estimates and gait symmetry measures with minimal bias and acceptable variability across both multi‐angle fixed and handheld setups.

## 2 Materials and Methods

### 2.1 Horses and Equipment

Eight horses were selected for the study from the teaching herd at the University of Copenhagen, Department of Veterinary Clinical Sciences. All the horses were familiar with exercise on the high‐speed treadmill. Local ethical approval was obtained in accordance with the university's ethical guidelines (the Local Ethical and Administrative Committee of the Department of Veterinary Clinical Sciences, no. 2024‐007). All horses were brown, Standardbred trotter mares with a weight range of 414–580 kg and a height range of 154–168 cm. They were not selected or screened based on lameness status. Prior to inclusion, each horse was trotted in hand on a hard surface and visually assessed by an experienced veterinarian (S.S.H.) to ensure it was fit for treadmill work, but no formal lameness examination was performed. The horses were trotted at an individual pace on the treadmill between 3 and 5 min, where the trot was uniform and stable, without pushing the horses forward.

**FIGURE 1:**
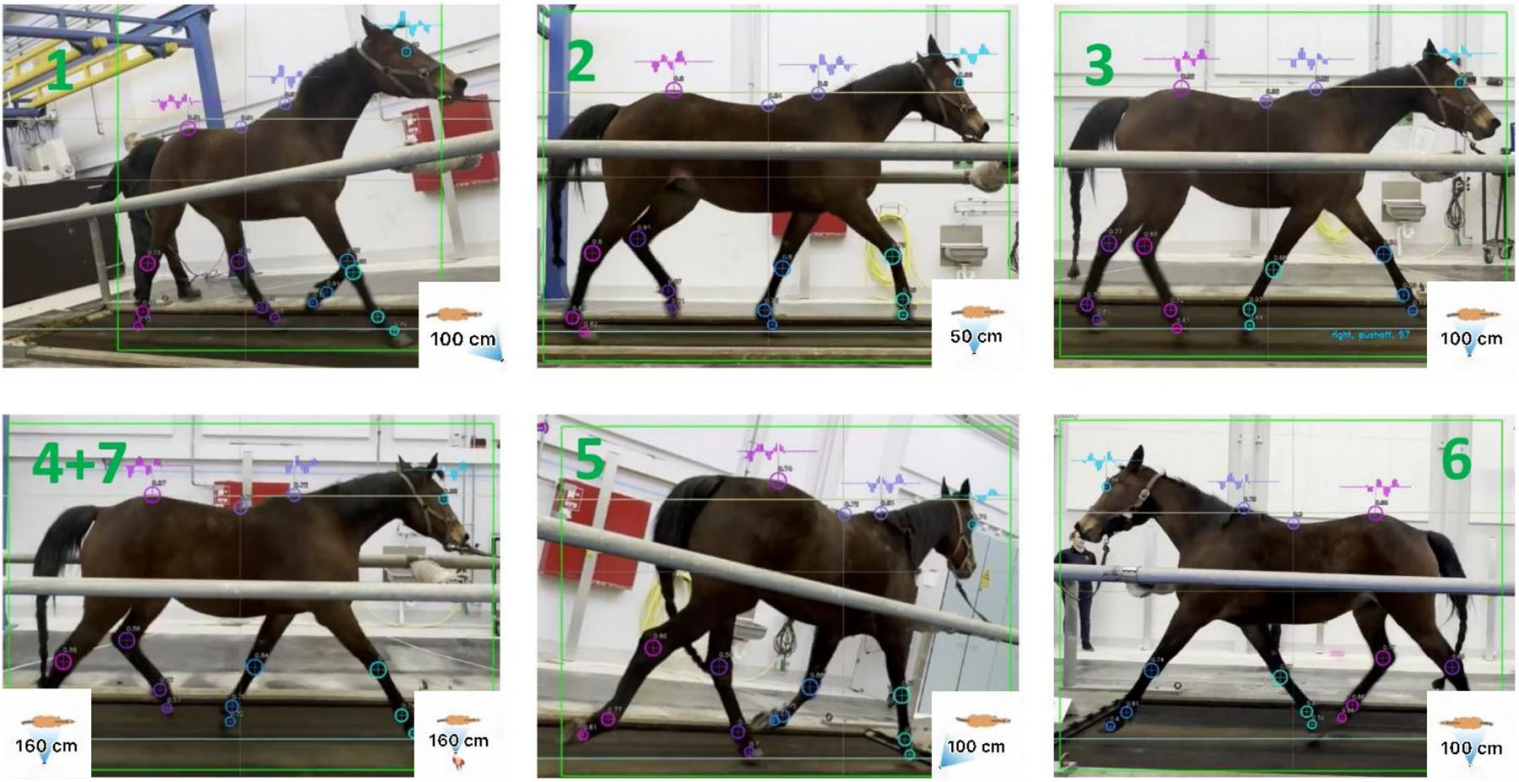
Camera positions and angles relative to the treadmill and horse. The dynamic estimated groundline is visible near the horse's hooves. The animations in the corners show the camera angle as seen from above.

During trot on the high‐speed treadmill, each horse was recorded by seven iPhones (Model 15 Pro, iOS‐Version 15.5.1, Apple Inc., Palo Alto, California, United States) positioned around the treadmill. The cameras were placed at various heights and angles to capture different perspectives of the horses' movement. The specific camera positions are outlined in Table 1 and Figure 1. The handheld camera was operated by the same person (K.K.), who aimed to walk on the spot approximately at 1 s stride intervals, creating clear camera movement. The treadmill was placed in a room with a limited distance from the horse of 300 cm on the right side and 250 cm on the left side. The average trotting speed was 4.8 m/s (4.5–5.2 m/s).

**TABLE 1:** Camera setup and positioning.

Camera	Position	Distance from midline treadmill (cm)	Angle (°)	Height (cm)
1	Oblique front right	300	50	100
2	Right side	300	90	50
3	Right side	300	90	100
4	Right side	300	90	160
5	Oblique rear right	300	50	100
6	Left side	250	90	100
7	Handheld right side	300	90	∼160

**FIGURE 2:**
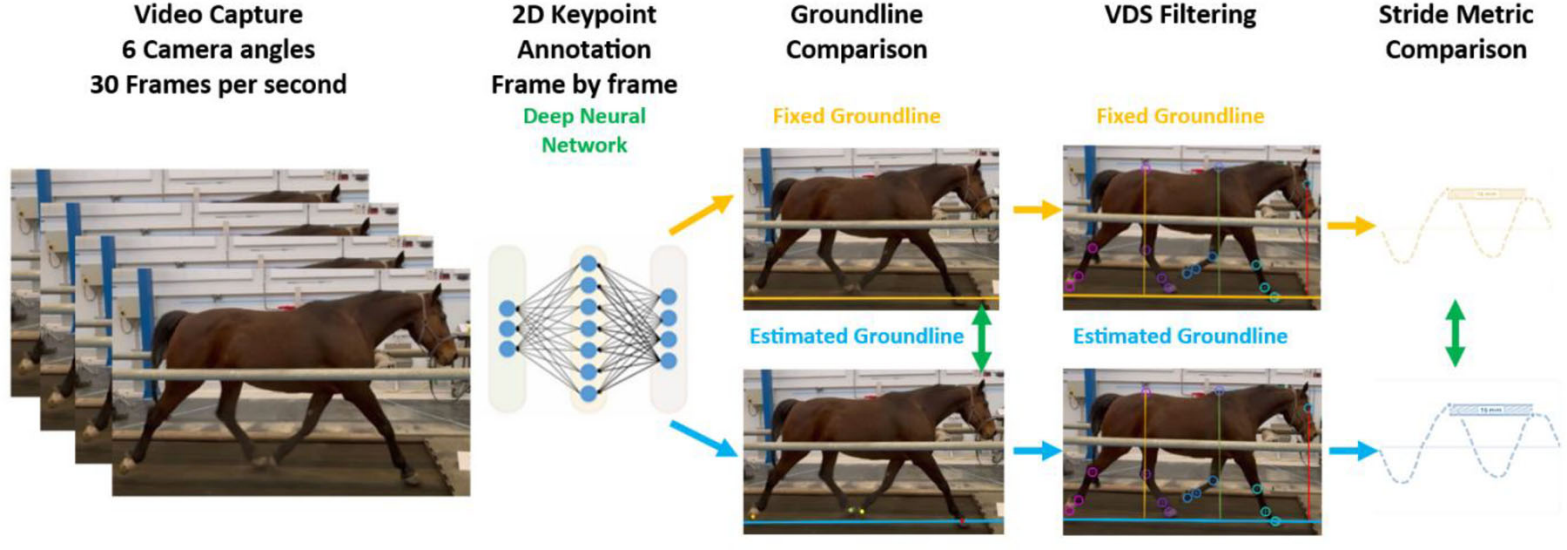
Experimental setup. The study population (*n* = 8) were recorded from six angles as described in Figure 1 with iPhones recording at HD, 30 FPS. The frames were keypoint‐annotated by a trained deep neural network. The groundline was either fixed or dynamically estimated by using the hoof keypoints, and the VDS were filtered. The resulting VDS signals were stride split, and the matching strides were compared statistically by comparing Mindiff and Maxdiff. The VDS curves on the right illustrate matching strides and a Maxdiff calculation as an example.

### 2.2 Data Collection

The horses were recorded with seven cameras (Figure 1), and videos were synchronized with one‐frame precision using a light source visible to all cameras. The recordings were captured using an ultrawide lens (13 mm, ƒ/2.2) in 4K at 60 Frames per second (FPS) and later converted to HD at 30 FPS before analysis, as this is the standard setting used by the algorithm (Figure 2). The ultrawide lens was selected to ensure that the pixel‐to‐metric scale matched real‐world applications.

**FIGURE 3:**
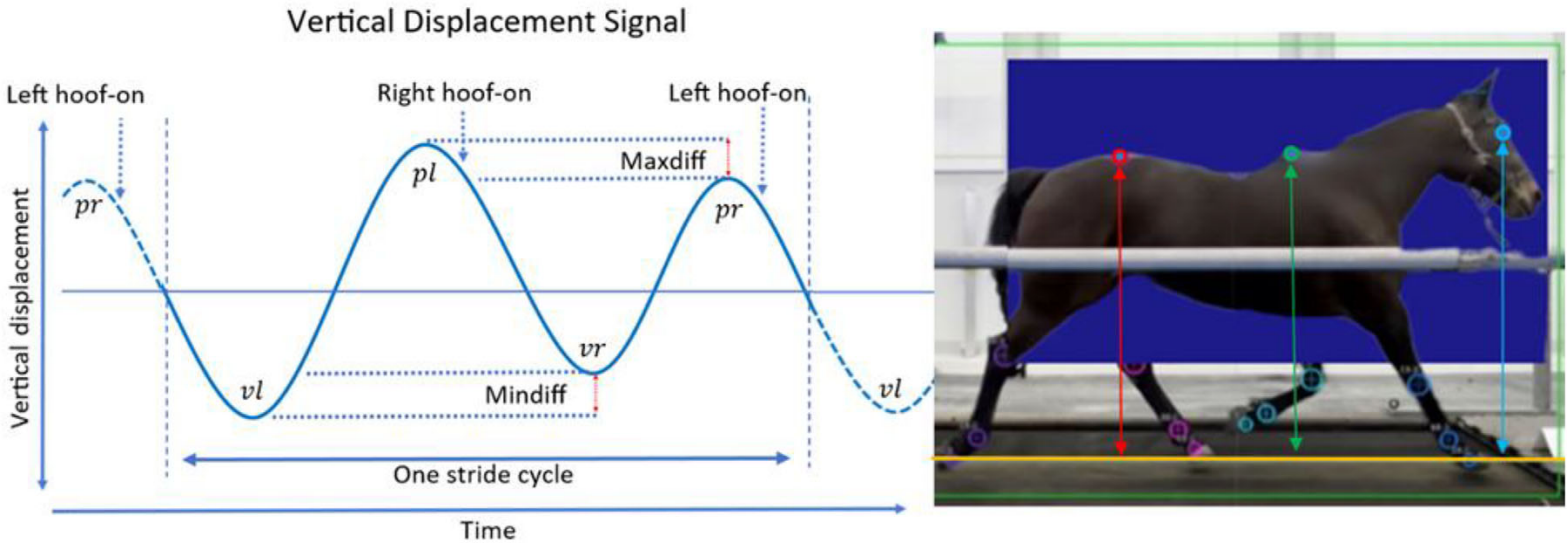
The vertical displacements (red, green, blue arrows) are measured in the 2D sagittal plane of the horse (blue square), when seen from the side, as the distance in pixels from the dynamically estimated groundline (orange) or a fixed groundline to the keypoints. The pixel difference is calibrated to a metric value, knowing the metric withers height.

The custom vision‐based algorithm (RealHorse, Keydiagnostics ApS, Fredensborg, Denmark) processed the video segments to estimate a frame‐by‐frame ‘dynamic groundline’ (as seen in Figures 1 and 3) based on hoof keypoints. The control dataset consisted of the exact same videos where the groundline was fixed by using the physical outline of the treadmill base (Figure 2). After this point, the two datasets had the same downstream data processing. The Vertical Displacement Signal (VDS) (Figure 3) relative to each groundline was calculated for three keypoints: eye, withers and croup (tuber sacrale). The data were then filtered and analysed by the algorithm without excluding any outlier strides. The algorithm computed the maximum (Maxdiff) and minimum (Mindiff) differences in VDS across all strides for each keypoint. Maxdiff and Mindiff values, derived using both the algorithm‐estimated groundline and a fixed groundline, were used for subsequent comparative analysis.

#### 2.2.1 Signal Filtering and Symmetry Metrics Computation

The core analysis relied on a trained deep neural network to identify keypoints on the horse from a side‐view video. These keypoints included the eye, withers, back and croup (tuber sacrale), as well as three points on each leg: hoof, metacarpo‐/metatarsophalangeal joints and carpus/tarsus. This model was trained on a large dataset (231 videos, 65,000 frames and over one million manually annotated keypoints) that included a diverse range of horse types trotting from a side perspective in straight lines, from the centre on the lunge and one horse on a treadmill.

The model processed each frame of the recorded video, identifying high‐confidence segments where all relevant keypoints were visible. This helped exclude segments unsuitable for analysis, such as moments when head movement obscured the eye keypoint, leading to minor variations in detected strides across cameras for the eye, withers and croup.

To standardize the reference frame, the vision‐based algorithm estimated a dynamic groundline based on hoof keypoints (Figures 1 and 3). A control dataset was created using the same videos but with a marked and fixed groundline. After marking the groundline, using the base of the treadmill, each frame was rectified using the same transformation (corresponding to the central frame) throughout the video. After this step, both datasets underwent identical downstream processing (Figure 2).

The VDS (Figure 3) was computed relative to each groundline for the eye, withers and croup. The recordings were calibrated using the known metric withers height of the horse, allowing vertical displacement to be expressed in millimetres. The raw VDS contained both motion‐related signals and noise from factors such as camera movement, keypoint imprecision and non‐periodic horse motions (e.g., sudden head movements affecting the eye's VDS).

To extract relevant frequency components, the expected stride frequency was estimated to account for variations in velocity. A 3rd‐order Butterworth high‐pass filter was applied, with a cut‐off set 5% below each horse’ algorithm estimated trot frequency, removing low‐frequency noise while preserving motion characteristics associated with trotting (Bosch et al. 2018; Serra Bragança et al. 2020). After filtering, individual strides were extracted based on leg keypoint information, including algorithm recognition of left versus right, with stride boundaries identified using expected maxima and minima (Rhodin et al. 2022).

The Maxdiff (maximum VDS difference) and Mindiff (minimum VDS difference) values for each stride were then computed (Keegan 2007; Keegan et al. 2011; Pfau et al. 2005; Hardeman, Egenvall et al. 2022; Roepstorff et al. 2021; Buchner et al. 1996; Rhodin et al. 2022; Starke and Clayton 2015; Serra Bragança et al. 2020). These values, derived using both the dynamic and fixed groundlines, were used for comparative analysis.

The videos from the eight horses were divided into trial‐like shorter segments of an average of 17 strides, creating a sample size of 357 trials, which were compared between handheld and stationary recording

### 2.3 Data Analysis

#### 2.3.1 Groundline Angle Error

In addition to vertical displacement metrics, we also evaluated the groundline angle error by comparing the vision‐based estimate to the known fixed groundline in all frames. Histograms of signed angle error and absolute angle error were created to reveal potential bias (mean error) and overall accuracy mean average error (MAE) in groundline orientation.

#### 2.3.2 Descriptive Statistics

For each stride and each of the keypoints eye, withers and croup, Maxdiff and Mindiff were extracted under both the fixed and the estimated groundline conditions. The number of valid strides included in the analysis varied slightly due to occasional frame‐level occlusions or detection errors, resulting in small differences in sample size across keypoints. The resulting data were pooled across all cameras and all horses, yielding large sample sizes for both Maxdiff and Mindiff comparisons. We computed mean differences, 95% limits of agreement (LoA), mean absolute differences (MAE) and 99% confidence intervals (CI) to provide an overall summary of measurement error. Histograms of differences provided a quick visual check for systematic over‐ or under‐estimation. Histograms of MAE further characterized how large the deviations were, irrespective of sign.

All statistical analyses were performed using Microsoft Excel (Version 2410, Build 16.0.18129.20158, 64‐bit) (Microsoft Corporation, Redmond, WA, USA).

#### 2.3.3 Scatter and Bland‐Altman Plots

Scatter plots of fixed versus estimated groundline measurements (Maxdiff or Mindiff) were generated to visualize how closely the values aligned.

To quantify agreement in more detail, Bland–Altman plots were generated for both Maxdiff and Mindiff, showing the difference between estimated and fixed measurements plotted against the mean of these two measurements. The mean difference and the 95% LoA were plotted to identify any systematic bias and examine whether the variance of the differences changed with the magnitude of the measured values. Outliers outside the range of the plot were noted in the figure text.

## 3 Results

### 3.1 Groundline Angle Evaluation

**FIGURE 4:**
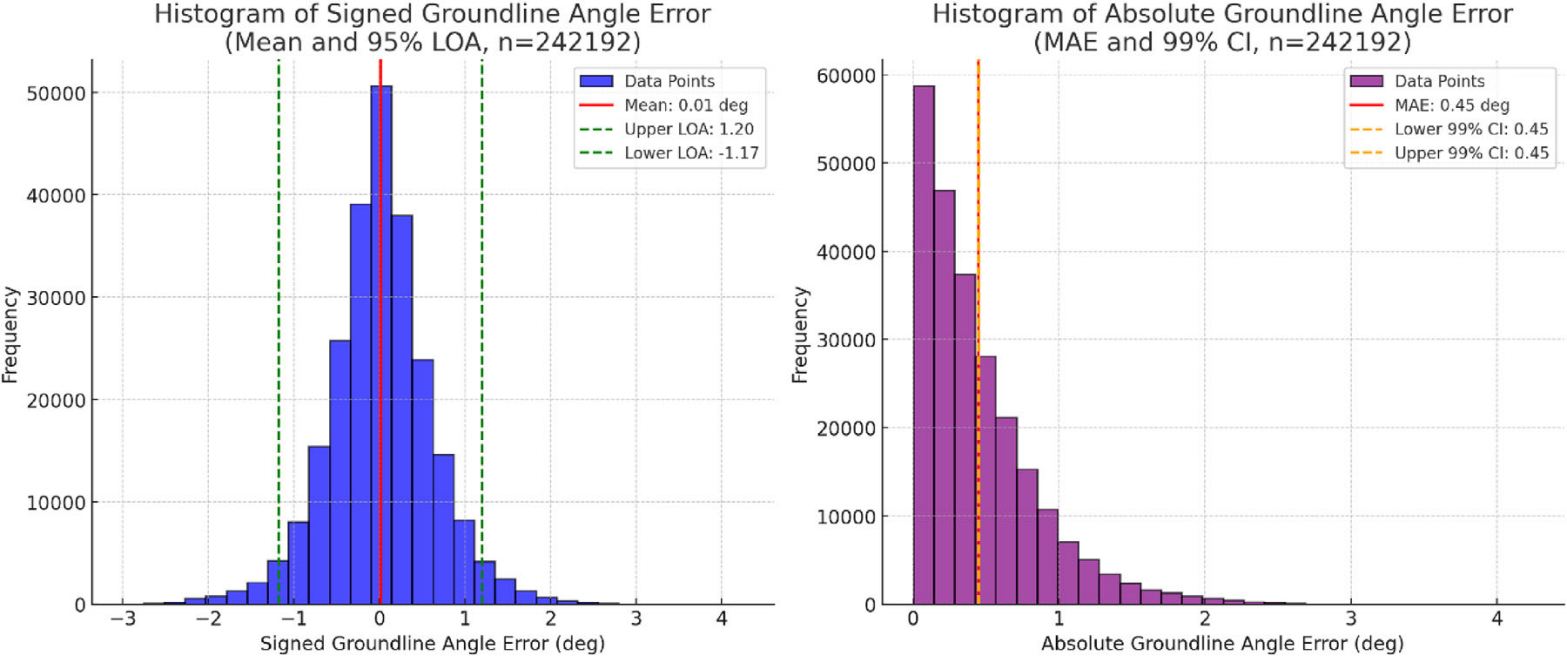
Histograms of signed groundline angle error (left) and absolute groundline angle error (right).

Figure 4 shows histograms of the signed and absolute groundline angle errors across all recorded frames (*n *= 242,192). The mean signed angle error was 0.01°, with 95% LoA ranging from −1.17° to +1.20°. The MAE of the groundline angle was 0.45° and the 99% CI fell within ±0.45°.

### 3.2 Stride‐Based Comparison Between Estimated and Fixed Groundlines

#### 3.2.1 Stride Detection and Missing Data

A total of eight horses were recorded, yielding a minimally varying number of detected strides after excluding occluded or incomplete data. The theoretical maximum number of detectable strides, estimated using the mean stride length, was 12,672. The actual detected stride sample sizes for stationary cameras 1–6 were as follows: eye: *n* = 12,256, withers: *n* = 12,462 and croup: *n* = 12,261.

This corresponds to a stride detection failure rate of 2%–3%.

#### 3.2.2 Mindiff Agreement

**FIGURE 5:**
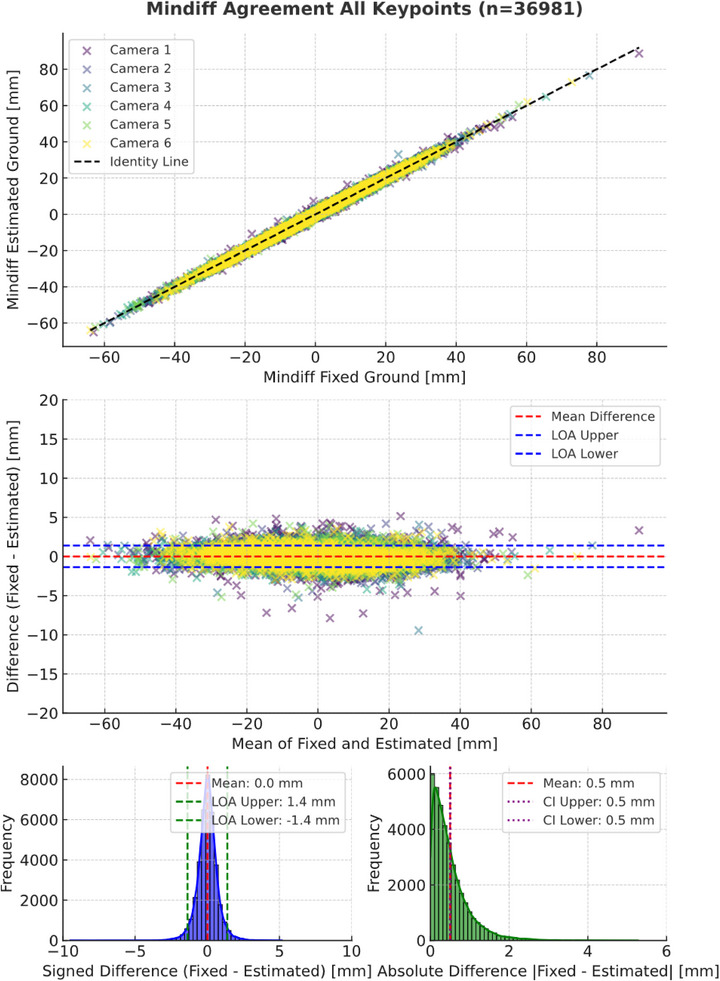
Scatter plot (top), Bland‐Altman plot (middle) and histograms of signed and absolute differences (bottom) for MinDiff values comparing estimated versus fixed groundlines across all keypoints.

For Mindiff, the dataset included 36,981 comparisons between fixed and estimated groundlines across all keypoints (eye, withers and croup) and strides. Figure 5 presents the scatter plot, Bland‐Altman plot and histograms of the differences. The MSE was −0.01 mm, with a 95% LoA of (−1.39 to +1.36) mm and the MAE was 0.50 mm, with a 99% CI of (0.50 to 0.51) mm.

#### 3.2.3 Maxdiff Agreement

**FIGURE 6:**
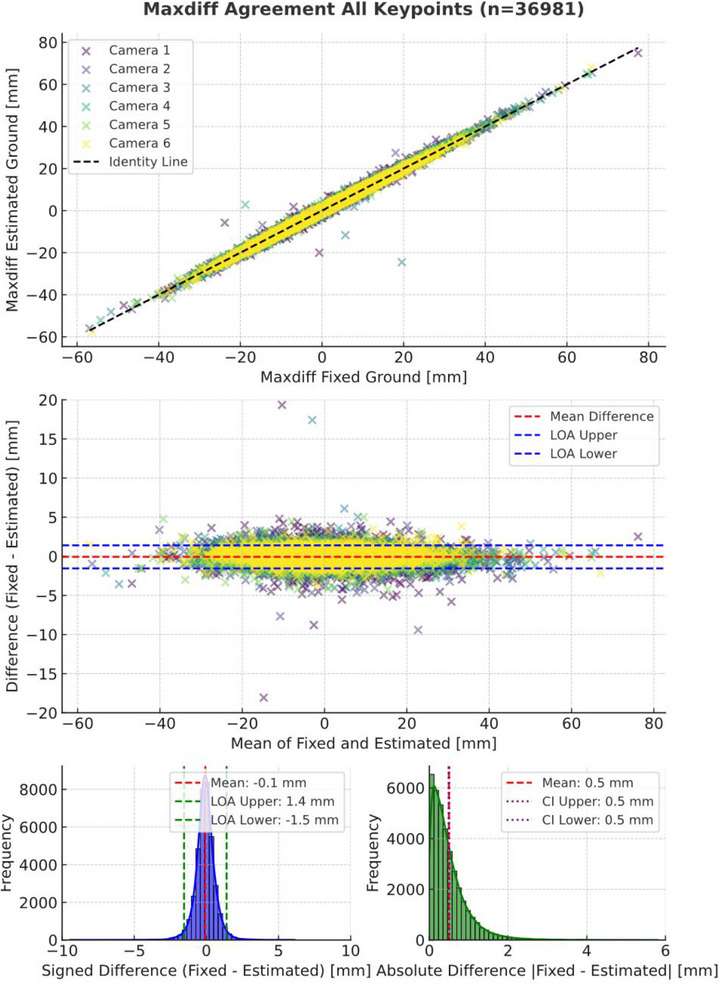
Scatter plot (top), Bland‐Altman plot (middle) and histograms of signed and absolute differences (bottom) for MaxDiff values comparing estimated versus fixed groundlines across all keypoints. Bland–Altman outliers outside plot: (19.6, −44.1; −18.8, 21.6).

For Maxdiff, a total of 36,979 stride comparisons were made, as illustrated in Figure 6. The mean signed difference was 0.07 mm, with a 95% LoA of (−1.31 to +1.46) mm and the MAE was 0.50 mm, with a 99% CI of (0.49 to 0.51) mm.

The scatter and Bland‐Altman plots show a strong correlation, with most data points tightly clustered around the line of identity and minimal systematic bias. Two outlier strides outside the chosen range of the Bland‐Altman plot were noted.

### 3.3 Comparison Between Handheld and Stationary Cameras

After excluding frames with missing keypoint detections, the number of successfully matched strides between the handheld camera (Camera 7) and the stationary camera (Camera 4) was: 2053 for the eye keypoint, 2081 for the withers and 2095 for the croup. These values represent the number of strides for which valid symmetry metrics could be computed for each anatomical region. Given a theoretical maximum of 2112 strides, the stride detection failure rate ranged from 1% to 3%, depending on the keypoint.

#### 3.3.1 Stride‐Based Results

Table 2 summarizes the comparison between handheld and stationary cameras for the keypoints eye, withers and croup. For both Maxdiff and Mindiff metrics, mean signed differences close to zero and narrow 95% LoA were observed for all keypoints.

**TABLE 2:** Stride‐based agreement between handheld and stationary cameras.

Keypoint	Metric	Mean signed error (mm)	95% LoA (mm)	Mean absolute error (mm)	95% CI of MAE (mm)
Eye	Maxdiff	0.18	−9.60 to +9.96	3.84	3.70 to 3.97
Eye	Mindiff	−0.27	−10.32 to +9.78	4.02	3.88 to 4.15
Wither	Maxdiff	0.33	−11.85 to +12.51	4.85	4.69 to 5.02
Wither	Mindiff	−0.02	−11.12 to +11.08	4.42	4.27 to 4.58
Croup	Maxdiff	−0.13	−9.73 to +9.47	3.86	3.73 to 3.99
Croup	Midkiff	−0.86	−10.53 to +8.80	3.98	3.85 to 4.11

**FIGURE 7:**
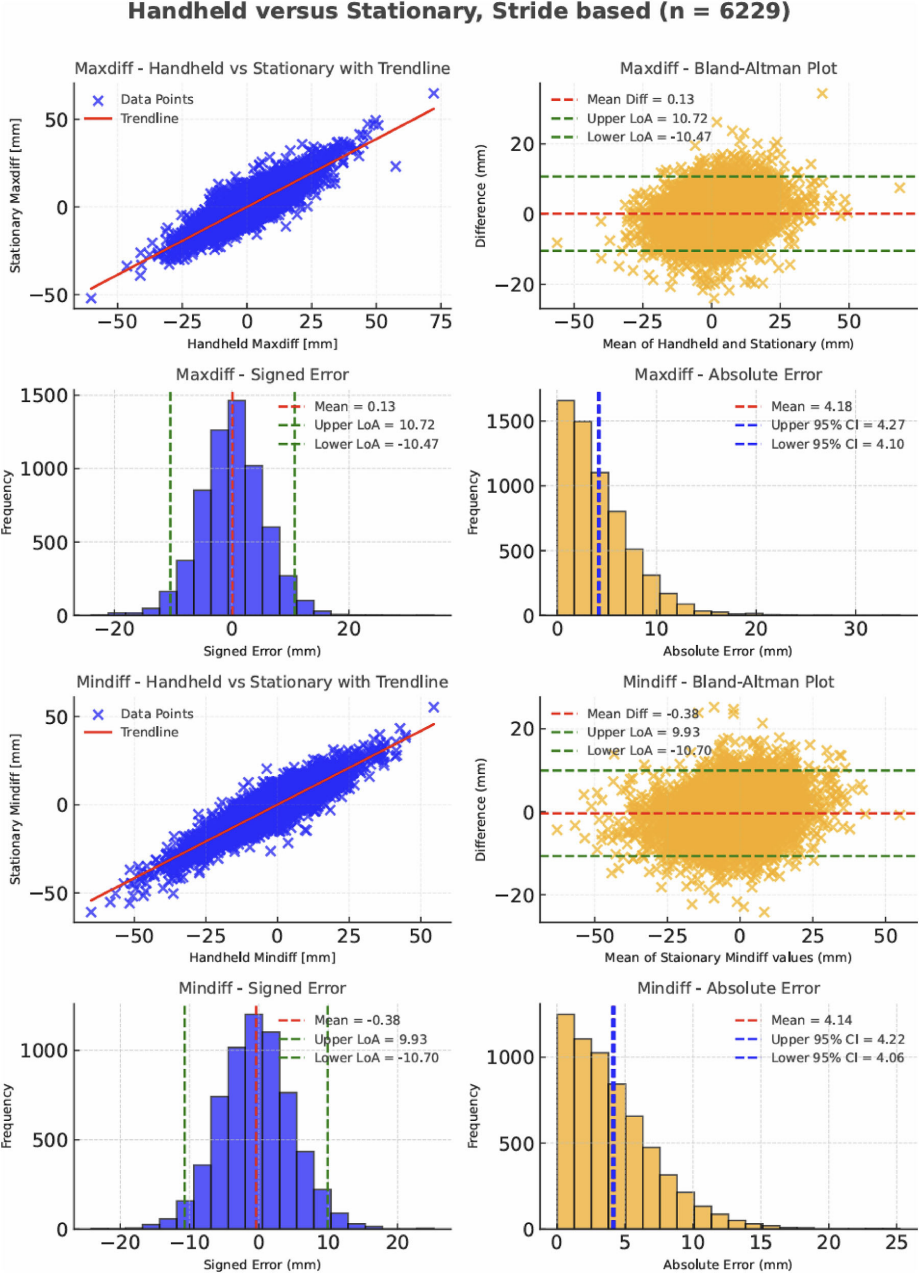
All keypoints—Stride‐based comparison between handheld and stationary camera. Scatter plot (top), Bland‐Altman plot (middle) and histograms of signed and absolute differences (bottom) for Maxdiff and Mindiff values comparing handheld versus stationary cameras across all keypoints (*n* = 6229).

Figure 7 illustrates the overall agreement for all three keypoints (*n* = 6229) with scatterplot, Bland‐Altman plot and histograms of signed and absolute differences. The Maxdiff 95% LoA ranged from −10.47 to +10.72 mm, with a MAE of 4.18 mm (95% CI: 4.10 to 4.27 mm). The Mindiff 95% LoA ranged from −10.70 to +9.93 mm, with a MAE of 4.14 mm (95% CI: 4.06 to 4.22 mm).

#### 3.3.2 Trial‐Based Results

**FIGURE 8:**
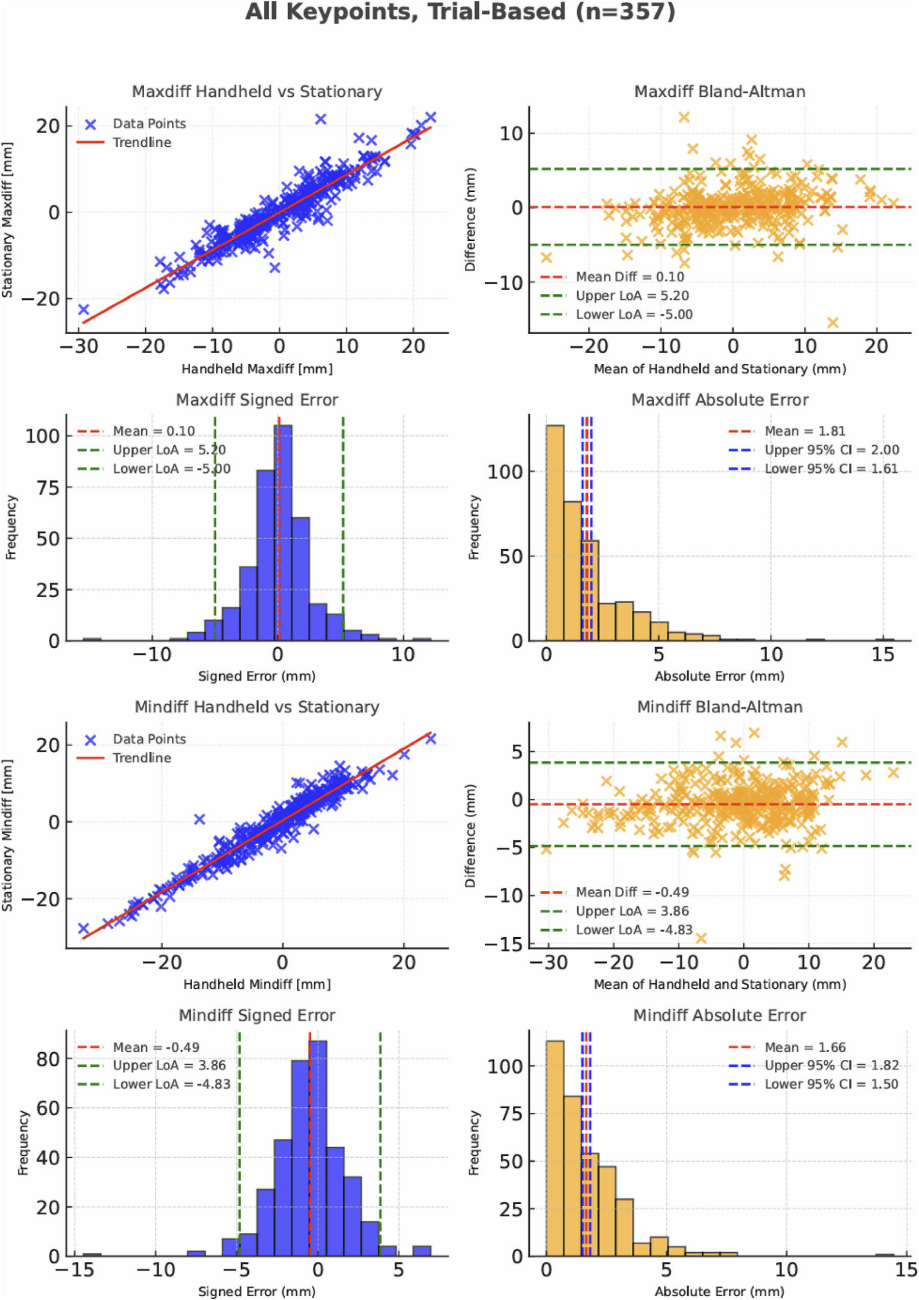
All keypoints—Trial‐based comparison between handheld and stationary camera. Scatter plot (left), Bland‐Altman plot (right) and histograms of signed and absolute differences (bottom) for Maxdiff and Mindiff values comparing handheld versus stationary cameras across all keypoints (*n* = 357).

A total number of 357 trials were compared between handheld and stationary recording (Table 3). The MAE per trial for all keypoints (Figure 8) were 1.8 mm (95% CI: 1.6 to 2.0 mm) and 1.7 mm (95% CI: 1.5 to 1.8 mm) for Maxdiff and Mindiff, respectively and considerably lower than the within‐stride MAE (Figure 7).

**TABLE 3:** Trial‐based agreement between handheld and stationary cameras.

Keypoint	Metric	Mean signed error (mm)	95% LoA (mm)	Mean absolute error (mm)	95% CI of MAE (mm)
Eye	Maxdiff	0.22	−3.27 to +3.72	1.30	1.07 to 1.54
Eye	Mindiff	−0.33	−3.76 to +3.09	1.38	1.17 to 1.59
Withers	Maxdiff	0.10	−6.17 to +6.37	2.33	1.95 to 2.71
Withers	Mindiff	−0.21	−5.19 to +4.77	1.74	1.42 to 2.06
Croup	Maxdiff	−0.00	−4.93 to +4.92	1.70	1.38 to 2.02
Croup	Mindiff	−0.89	−5.14 to +3.35	1.81	1.55 to 2.07
All keypoints	Maxdiff	0.10	−5.00 to +5.20	1.81	1.61 to 2.00
All keypoints	Mindiff	−0.49	−4.83 to +3.86	1.66	1.50 to 1.82

## 4 Discussion

In the present study, we investigated how a vision‐based algorithm performs when automatically estimating the groundline compared to a fixed groundline reference, under controlled circumstances with horses trotting on a treadmill. We evaluated the consistency of groundline angle error and stride‐based Maxdiff and Mindiff across a range of camera angles and heights, including handheld use, which is especially relevant for clinical and field applications.

The algorithm exhibited a low failure rate, with only a small number of strides failing to be analysed due to missing keypoint detection in a frame of the strides; this highlights a robustness in handling challenging camera angles.

One of the objectives was to quantify how reliably the vision‐based system could estimate a groundline compared to a fixed horizontal reference. Across more than 240,000 frames, the algorithm showed a mean signed angle error of only 0.01°, with 95% LoA ranging from 1.17° to +1.20°. The MAE was 0.45 °, with a 99% CI interval within ±0.45°. These data suggest that, despite stress‐testing with varying camera heights and angles, the system consistently detects ground orientation with minimal bias. Previous methods for gait analysis often require markers or additional calibration objects to establish a precise ground reference (Keegan et al. 2011; Roepstorff et al. 2021). In contrast, the current approach leverages automated keypoint tracking of the limbs to derive a frame‐by‐frame continuous, ‘dynamic groundline’. This approach may simplify clinical setups by eliminating the need for manual calibration and allowing for greater flexibility in real‐world conditions.

An accurate groundline is crucial for any ground truth method of measuring stride‐based metrics, such as peak (Maxdiff) and lowest (Mindiff) vertical displacement differences, which are widely used for lameness and symmetry assessments in horses (Keegan 2007; Pfau et al. 2005). When comparing the estimated versus fixed groundline, both Maxdiff and Mindiff metrics showed near‐zero mean differences (± 0.1 mm) and a narrow mean absolute error (∼0.5 mm). The 95% LoA spanned approximately ± 1.4 mm, indicating that automated groundline estimation introduces minimal additional error and no bias into the VDS. From a clinical perspective, differences of a few millimetres in Maxdiff or Mindiff are within the range of measurement noise in most gait analysis tools (Keegan et al. 2011). Accordingly, using the algorithm‐estimated groundline appears to be a reliable alternative to traditional manual methods, facilitating accurate stride‐by‐stride analysis under controlled circumstances (horses trotting on a treadmill). The many different camera angles and perspectives in this study were aimed to stress‐testing the algorithm to better simulate real‐world applications.

Handheld use appeals to both clinicians and horse owners, and it is essential for this system, as the algorithm relies on side‐view recordings of horses trotting in a straight line or from the centre of the circle when being lunged. However, handheld recording inherently introduces camera movement. When evaluating the algorithm's performance under handheld use, we found that it maintained accuracy, with only small biases observed.

Stride‐based Maxdiff and Mindiff comparison between a handheld camera and a stationary reference camera showed small biases (close to zero) and a narrow 95% LoA (within ±10 to 12 mm for the eye, withers and croup). The MAE remained well under 5 mm, indicating that handheld camera motion did not substantially degrade VDS calculation. When averaged at the trial level, typically consisting of ∼17 strides, the measurement error was even lower, with eye and croup below 1.8 mm.

While this study demonstrated that the vision‐based algorithm accurately estimates the groundline and computes reliable stride‐level symmetry metrics under controlled conditions, several limitations should be acknowledged. The use of a treadmill, a relatively small and homogeneous sample of horses of the same breed and coat colour, and the lack of subjective lameness assessment may limit generalizability. Further research is needed to expand its clinical applicability. Future work should validate the algorithm in overground settings with uneven surfaces, variable lighting and diverse camera angles. Including a broader range of breeds, coat colours and movement patterns would help assess generalizability across the equine population. Additionally, comparison with established 3D motion capture or inertial sensor systems would provide an external reference standard for absolute accuracy. Finally, integrating the algorithm into real‐time or near‐real‐time workflows may support its use in routine clinical practice and remote lameness monitoring.

## 5 Conclusion

This study demonstrated that a custom vision‐based algorithm (RealHorse) can accurately estimate the groundline and compute comparable stride‐based symmetry measures in trotting horses under controlled circumstances, across a range of stationary camera positions and a handheld setup. The method produced high‐accuracy VDS with negligible differences relative to a fixed ground reference. The minimal additional noise from handheld recordings highlights the system's flexibility, which is relevant for real‐world clinical applications.

While promising, further testing is needed to validate this approach in varied environments, different lighting conditions, different breeds and coat colours. Comparative assessments against established gait analysis systems would provide an external standard for accuracy. Nevertheless, these results support marker‐less smartphone‐based gait analysis as a practical, accessible technology for objective lameness detection and broader equine locomotor evaluations.

## Author Contributions

**Karsten Key**: conceptualization, writing – original draft, data curation, formal analysis, methodology. **Katja Berg**: writing – review and editing, data curation, methodology. **Jakob Kirkegaard**: software, data curation, writing – review and editing, methodology, formal analysis. **Kristian Ringkjær Andresen**: writing – review and editing. **Sabrina Skov Hansen**: writing – review and editing; data curation.

## Funding

The authors have nothing to report.

## Ethics Statement

Local ethical approval was obtained in accordance with the university's ethical guidelines (The Local Ethical and Administrative Committee of the University of Copenhagen, Department of Veterinary Clinical Sciences, no. 2024‐007).

## Consent

The author has nothing to report.

## Conflicts of Interest

Authors K.K., K.B. and J.K. are affiliated with Keydiagnostics ApS, a company that provides a commercially available smartphone application ‘RealHorse’ for detecting asymmetry in horses. The computer vision algorithm developed and tested in this study is part of this product. These affiliations may represent a potential conflict of interest, which is hereby disclosed.

## Data Availability

The data that support the findings of this study are openly available at EvaluatingtheaccuracyofaVision‐BasedAlgorithmforGroundlineEstimationinTrottingHorsesUsingMultipleCameraAngles.
